# Multi-epitope vaccine candidate design for dengue virus

**DOI:** 10.6026/97320630019628

**Published:** 2023-05-31

**Authors:** A Dharani, DR Ezhilarasi, G Priyadarsini, PA Abhinand

**Affiliations:** 1 Department of Bioinformatics, Sri Ramachandra Institute of Higher Education and Research, Porur, Chennai - 600 116, India

**Keywords:** Dengue, linkers, epitope, antigen, antibody, docking, immune simulation

## Abstract

Dengue Fever (DF) is a vector-borne neglected viral disease with a high burden in the sub-tropics of Asia and Africa. Aedes aegypti is responsible for 90% of cases in the global burden of disease. The primary goal of the treatment is to eliminate the virus from the bloodstream of affected individuals. A successful dengue vaccine must elicit both neutralizing antibodies and cell-mediated immunity and there is no vaccine to date to prevent DF. A multi-epitope vaccine composed of a series of or overlapping peptides is, therefore, an ideal approach for the prevention and treatment of pathogenic organisms. An immunoinformatics approach was employed to design a theoretical multi-epitope vaccine candidate. This vaccine candidate consists of linear B-cell epitope, TH cells epitope and CTL of reported potential vaccine candidates. These epitopes were linked together with suitable linkers and adjuvant at the N terminal and C terminal. The 3D Structure of the vaccine was modeled, refined and validated using computational tools. Protein-protein docking of vaccine candidates with TLR3 protein results in efficient binding. Immune stimulation of vaccine candidates predicted high levels of IgG and IgM. This candidate vaccine should be validated experimentally using suitable in-vivo and in-vitro studies to use in dengue fever virus elimination programmes.

## Background:

Dengue fever is a mosquito-borne tropical disease caused by the dengue virus. Dengue virus is transmitted by female mosquitoes mainly of the species Aedes aegypti and, to a lesser extent, Ae. albopictus. Dengue has become a global burden since the Second World War, mainly in South Asia. About 390 million people are infected a year and approximately 25,000 people die [1]. Dengue fever virus (DENV) is a single positive-stranded RNA virus of the family Flaviviridae, genus Flavivirus. Dengue viruses consist of four antigenically related but distinct DENV serotypes (DENV-1, DENV-2, DENV-3, and DENV-4) [2]. The female Aedes aegypti mosquito becomes a vector or carrier of the virus when it bites an infected human. When a mosquito feeds on human blood, it picks up the virus as part of its blood meal. This virus can survive in the mosquito's gut for up to ten days, after which period it can spread the infection to any other individual it bites and feeds on [3]. DF occurs as a result of both primary and secondary infections, and it is most common in adults and older children. A biphasic, high-grade fever that lasts 3 days to 1 week precedes the onset of symptoms. Other symptoms include severe headache, lassitude, myalgia, and sore joints, as well as metallic taste, appetite loss, diarrhoea, vomiting, and stomach ache. DHF is a common complication of a secondary dengue infection. However, due to maternally acquired dengue antibodies, it may also happen during a primary infection in infants. The clinical course of DHF is divided into three stages: febrile, leakage, and recovery. DHF with an unstable pulse, narrow pulse pressure (20 mmHg), restlessness, cold, clammy skin, and circumoral cyanosis is classified as DSS. The high mortality rate associated with DSS is due to progressive worsening shock, multiorgan injury, and disseminated intravascular coagulation. According to the World Health Organization (WHO), the dengue cases increased over 8 fold over the last 2 decades, from 5.5lakhs cases in 2000 to 2.4 million in 2010 and to 4.2 million in 2019.In India, approximately 1.2 lakhs people are affected every year. Unfortunately, the highest number of cases and deaths were reported in 2017 [4]. It was One lakh eighty-eight thousand cases and three hundred twenty-five deaths (1, 88,000 cases & 325 deaths) and in Tamil Nadu Twenty-three thousand two hundred ninety-four cases and sixty-five deaths (23294 cases & 65 deaths). Thus, a preventive vaccine to dengue has been a priority for the WHO agenda for several decades. DF treatment is symptomatic. There is no specific treatment for dengue fever. During the acute period, bed rest is recommended. Antipyretics are medications that are used to reduce body temperature. Patients with heavy sweating or vomiting should receive oral fluid and electrolyte therapy. Available licensed dengue vaccine is not efficacious against South Asia dengue serotypes [5]. Successful dengue vaccine must elicit both neutralizing antibodies and cell mediated immunity. Preventative and therapeutic dengue vaccines are needed as long-term solutions. Epitope-based vaccines represent a novel approach for generating a specific immune response and avoiding responses against other unfavourable epitopes (like epitopes that may drive immunopathogenic or immune modulating responses) in the complete antigen. Potential advantages of epitope-based vaccines also include increased safety, the opportunity to rationally engineer the epitopes for increased potency and breadth, and the ability to focus immune responses on conserved epitopes. This work therefore focussed on the in-silico design and development of a potential multi-epitope vaccine peptide for dengue fever virus using E protein sequence. To design a multi-epitope vaccine against DENV using Immunoinformatics and Structural Bioinformatics approach.

## Methodology:

## Protein selection for vaccine:

**Figure 1:**
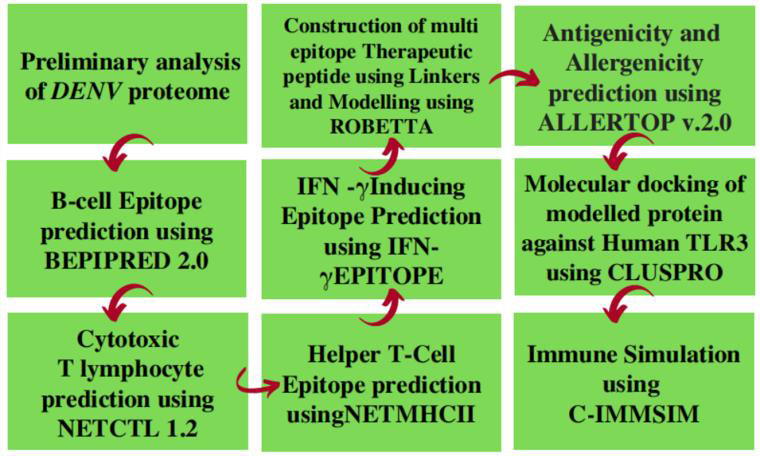
Workflow

Proteins were taken from UniProtKB: The UniProt Knowledgebase (UniProt ID: A0A2P0X374). The overall workflow of this study is given in Figure 1.

## Linear B-cell epitope prediction:

BepiPred-2.0 is a web server for predicting B-cell epitopes from antigen sequences. It is based on a random forest algorithm trained on epitopes annotated from antibody-antigen protein structures. This new method was found to outperform other available tools for sequence-based epitope prediction both on epitope data derived from solved 3D structures and on a large collection of linear epitopes downloaded from the IEDB [6].

## Cytotoxic T-lymphocyte (CTL) epitope prediction:

NetCTL is a web-based tool designed for predicting CTL epitopes. It does so by integrating predictions of proteasomal cleavage, TAP transport efficiency, and MHC class I affinity. At least four other methods have been developed recently that likewise attempt to predict CTL epitopes: EpiJen, MAPPP, MHC-pathway and WAPP. In order to compare the performance of prediction methods, objective benchmarks and standardized performance measures are needed. NetCTL works with large-scale benchmarks and corresponding performance measures and reports the performance of an updated version 1.2 of NetCTL in comparison with the four other methods. The prediction method integrates prediction of MHC class I binding peptides, proteasomal C-terminal cleavage, and TAP (Transporter Associated with Antigen Processing) transport efficiency. Though the server allows for predictions of CTL epitopes restricted to 12 MHC class I super types [7].

## T helper cells epitope prediction:

There are two versions of MHC-II-peptide binding affinity prediction methods, NetMHCII and NetMHCIIpan. These were constructed using an extended data set of quantitative MHC-peptide binding affinity data obtained from the Immune Epitope Database covering HLA-DR, HLA-DQ, HLA-DP and H-2 mouse molecules using artificial neural networks. The prediction of MHC II epitopes was based on receptor affinity, which can be inferred from the IC50 values and percentile ranks assigned to each predicted epitope. High-affinity peptides should have IC50 values <50nM. An IC50 value <500nM indicates intermediate affinity, while values <5000nM indicate low affinity.

## Construction of Multi epitope Vaccine Candidate Sequence:

**Figure 2:**
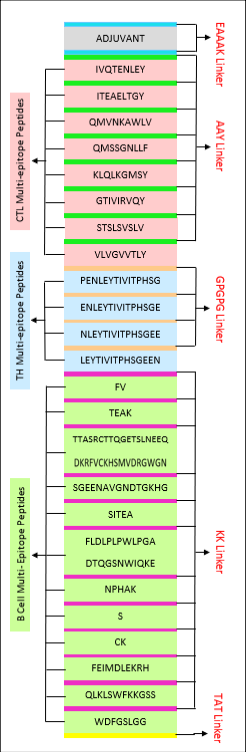
Schematic Representation of the Final Multi Epitope Vaccine

Predicted linear B-cell epitopes, high scoring CTL epitopes and high affinity TH epitopes were used to construct multi-epitope vaccine candidate sequence. The TH and CTL epitopes were linked using GPGPG [8] and AAY [9] linkers respectively and B cells are linked with KK [10]. To increase the vaccine immunogenicity, the β-defensin (45 mer) amino acid sequence was adjoined to the N-terminal with EAAAK linker of the vaccine [11]. The β-defensin peptides provoke innate immunity cells and recruit naive T cell through the chemokine receptor-6 (CCR-6) GIINTLQKYYRVRGGRAVLSLPKEEQIGKSTRGRKCRRKK. And at the c-terminal TAT was added to enable the intracellular delivery of the modeled vaccine [12]. The construction of multi-epitope vaccine is represented in Figure 2.

## Antigenicity and Allergenicity prediction:

ANTIGENpro is a sequence-based, alignment-free, and pathogen-independent predictor of protein antigenicity. The predictions are made by a two-stage architecture based on multiple representations of the primary sequence and five machine learning algorithms. A final SVM classifier summarizes the resulting predictions and predicts if the protein is likely to be antigenic or not as well as the corresponding probability. ANTIGENpro is the first predictor of the whole protein antigenicity trained using reactivity data obtained by protein microarray analysis for five pathogens. AllerTop v.2.0 reported a set of novel models for allergen prediction utilizing amino acid E-descriptors, auto- and cross-covariance transformation, and several machine learning methods for classification, including logistic regression (LR), decision tree (DT), naïve Bayes (NB), random forest (RF), multilayer perceptron (MLP) and k nearest neighbour (kNN). The best performing method was kNN with 85.3 % accuracy at 5-fold cross-validation. The resulting model has been implemented in a revised version on the AllerTop server [13].

## Physiochemical property prediction:

Various physicochemical properties for the designed protein were predicted using web server ProtParam (https://web.expasy.org/protparam/). ProtParam is a tool which allows the computation of various physical and chemical parameters for a given protein stored in Swiss-Prot. The computed parameters include the molecular weight, theoretical pI, amino acid composition, atomic composition, extinction coefficient, estimated half-life, instability index, aliphatic index and grand average of hydropathicity (GRAVY).

## Secondary structure prediction:

Self-optimized prediction method (SOPM) has been described to improve the success rate in the prediction of the secondary structure of proteins. SOPMA was used for secondary structure prediction of vaccine sequences.

## Tertiary structure modelling:

Ab initio- or de novo- protein modelling methods seek to build three-dimensional protein models "from scratch", i.e., based on physical principles rather than (directly) on previously solved structures. Robetta is a server that provides automated protein structure prediction and analysis capabilities. Sequences are processed into putative domains and structural models are constructed using either comparative modeling or de novo structure prediction approaches for structure prediction. If BLAST, PSI-BLAST, or 3D-Jury finds a confident match to a known structural protein, it is used as a template for comparative modelling. If no match is identified, the de novo Rosetta fragment insertion approach is used to make structural predictions. Data from experimental nuclear magnetic resonance (NMR) constraints can also be submitted using a RosettaNMR de novo structure query sequence. Other existing capabilities include predicting the effects of mutations on protein-protein interactions using the computational interface alanine scanning. In the near future, Rosetta's protein design and protein-protein docking technologies will be made available via the server [14].

## Molecular Docking using ClusPro:

CLUSPRO server provides a simple home page for basic use, requiring only two files in Protein Data Bank format. However, ClusPro also offers a number of advanced options to modify the search that include the removal of unstructured protein regions, applying attraction or repulsion, accounting for pairwise distance restraints, constructing homo-multimers, considering small angle X-ray scattering (SAXS) data, and finding heparin binding sites. Six different energy functions can be used depending on the type of proteins. Docking with each energy parameter set results in ten models defined by centres of highly populated clusters of low energy docked structures. This protocol describes the use of the various options, the construction of auxiliary restraints files, the selection of the energy parameters, and the analysis of the results. Although the server is heavily used, runs are generally completed in < 4 hours [15].

## Immune Simulation:

To further characterize the immunogenicity and immune response profile of multi-epitope vaccine candidates, in silico immune simulations were conducted using the C-ImmSim server (http://150.146.2.1/C-IMMSIM/index.php?page=1). C-ImmSim is an agent-based model that uses a position-specific scoring matrix (PSSM) for immune epitope prediction and machine learning techniques for prediction of immune interactions. It "simultaneously simulates three compartments that represent three separate anatomical regions found in mammals: (i) the bone marrow, where hematopoietic stem cells are simulated and produce new lymphoid and myeloid cells; (ii) the thymus, where naive T cells are selected to avoid auto immunity; and (iii) tertiary lymphatic organ, such as a lymph node".

## Result and Discussion:

## The choice of protein target:

The complete amino acid sequences of the E protein of Denv type 2 was retrieved from UniProtKB (https://www.uniprot.org/) in FASTA format. E protein, the smallest outer surface protein from Denv type 2 genome, was found to possess the highest antigenicity and is therefore used to identify B-cell and T-cell epitopes. E-protein can therefore be targeted as a potential vaccine target against Denv type 2 [16].

## Preliminary sequence analysis of the proteome of DENV:

Epitope-based vaccines represent a novel approach for generating a specific immune response and avoiding responses against other unfavourable epitopes (like epitopes that may drive immunopathogenic or immune modulating responses) in the complete antigen. This work therefore focused on the in-silico design and development of a potential multi-epitope vaccine peptide for dengue fever virus using E protein sequence. The dengue viral E protein is responsible for cell receptor binding and it is the main target for neutralizing antibodies. Therefore, the dengue E protein is an important antigen for vaccine development and diagnostic purposes.

## Prediction of multiple epitopes from the chosen immunogenic proteins:

Selected protein sequences are subjected to B cell prediction (BepiPred-2.0 web server) [17], Cytotoxic t lymphocytes prediction (NetCTL 1.2 server) and TH prediction (NetMHCII 2.3) [18]. The predicted peptide sequences containing linear B-cell epitopes and T-cell epitopes were fused using GPGPG and AAY linkers. a chemokine cell receptor was added. To the amino terminus of the vaccine peptide using an EAAAK linker in order to potentiate antigen-specific immune responses with Beta defensin in order to provoke innate immunity cells and recruit naive T cells through the chemokine receptor -6 [19]. In addition, a TAT sequence was added at the C-terminal to enable the intercellular delivery of the vaccine [12]. The final vaccine peptide generated consisted of 375 amino acid residues. Predicted probability of antigenicity with the viral model at threshold of 0.5 was found to be 0.872581 (ANTIGENpro) and it is predicted to be non-allergenic (AllerTOP 2.0)

## Prediction of tertiary structure models of the designed chimeric protein:

**Figure 3:**
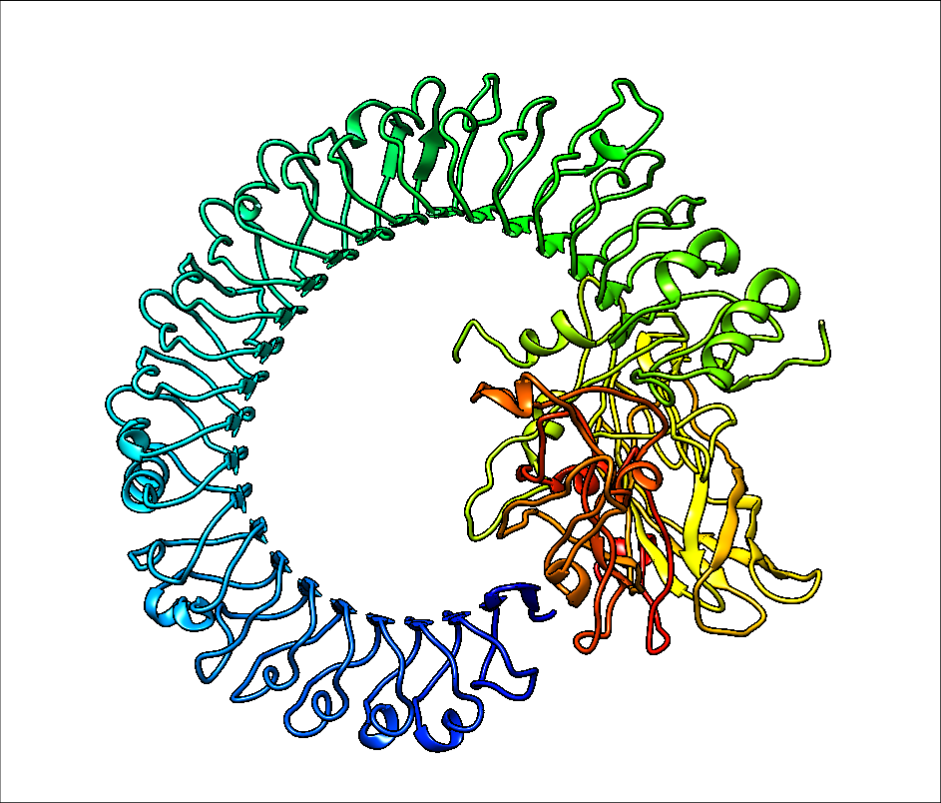
Docking complex of designed multi-epitope vaccine with Human TLR3 receptor (PDB ID: 2A0Z)

Ab-initio model for multi-epitope vaccine construct was modeled using robetta server. It gave five models as a result. The 3D structure of the vaccine candidate improved markedly after the refinement and showed desirable properties based on Ramachandran plot predictions. The Ramachandran plot shows 80.4% in most favoured regions, 0.9% in generously allowed and 0.3% disallowed regions; this indicates that the quality of the overall model is satisfactory. Protein-protein docking was performed and it shows good binding affinity with TLR3 (PDB ID: 2A0Z) was performed using cluspro 2.0. The predicted tertiary structure is given in Figure 3.

## Immune simulation of the designed Multi-Epitope Vaccine:

The docked complex model 1 has the lowest energy score (-1078.0kJ/mol) the highest binding affinity, thus was selected as the best-docked complex. Immune simulation showed results consistent with typical immune responses. C-ImmSim server immune simulation yielded results consistent with actual immune responses. In denv IgG and IgM responses to viral antigens are implicated in disease protection. The development of memory B-cells and T- cells was evident, with memory in B-cells lasting several months. Helper T cells were particularly stimulated. Neutralizing monoclonal antibodies is designed by using these linear and conformational epitopes against dengue virus that can work as an effective vaccine to save many precious lives.

## Conclusion:

The elimination of Dengue fever virus will not be achieved without novel control methods. These involve diagnostic and therapeutic tools as well as a vaccine if possible. In this study, immunoinformatics tools were employed to design a potential vaccine peptide coding for multiple B-cell and T-cell (TH and CTL) epitopes. Given that the proteins containing these epitopes could potentially provide both prophylactic and therapeutic benefits. This vaccine peptide could potentially be used as a complementary tool to achieve dengue fever virus elimination with further suitable in vivo and in vitro studies.
